# In Situ Test and Numerical Analysis of the Subway-Induced Vibration Influence in Historical and Cultural Reserves

**DOI:** 10.3390/s24092860

**Published:** 2024-04-30

**Authors:** Jie Su, Xingyi Liu, Yuzhe Wang, Xingyu Lu, Xiaokai Niu, Jiangtao Zhao

**Affiliations:** 1 Key Laboratory of Urban Underground Engineering of Ministry of Education, Beijing Jiaotong University, Beijing 100044, China; 19125870@bjtu.edu.cn (X.L.); wangyuzhe1@sxgkd.edu.cn (Y.W.); 22121122@bjtu.edu.cn (X.L.); 2 Shanxi Vocational University of Engineering Science and Technology, Jinzhong 030600, China; 3 Beijing Municipal Engineering Research Institute, Beijing 100037, China; metronxk@126.com (X.N.); sjszjtao@126.com (J.Z.)

**Keywords:** subway vibration, in situ test, spectrum analysis, dynamic coupling, vibration reduction scheme, periodic pile row

## Abstract

Although the rapid expansion of urban rail transit offers convenience to citizens, the issue of subway vibration cannot be overlooked. This study investigates the spatial distribution characteristics of vibration in the Fayuan Temple historic and cultural reserve. It involves using a V001 magnetoelectric acceleration sensor capable of monitoring low amplitudes with a sensitivity of 0.298 V/(m/s^2^), a measuring range of up to 20 m/s^2^, and a frequency range span from 0.5 to 100 Hz for in situ testing, analyzing the law of vibration propagation in this area, evaluating the impact on buildings, and determining the vibration reduction scheme. The reserve is divided into three zones based on the vertical vibration level measured during the in situ test as follows: severely excessive, generally excessive, and non-excessive vibration. Furthermore, the research develops a dynamic coupling model of vehicle–track–tunnel–stratum–structure to verify the damping effect of the wire spring floating plate track and periodic pile row. It compares the characteristics of three vibration reduction schemes, namely, internal vibration reduction reconstruction, periodic pile row, and anti-vibration reinforcement or reconstruction of buildings, proposing a comprehensive solution. Considering the construction conditions, difficulty, cost, and other factors, a periodic pile row is recommended as the primary treatment measure. If necessary, anti-vibration reinforcement or reconstruction of buildings can serve as supplemental measures.

## 1. Introduction

Train-induced vibration is indeed a significant issue, especially for buildings adjacent to the subway. In Beijing, a metropolis with a rich history, the metro line will inevitably pass by certain structures having historical and cultural significance [1]. Consequently, the safety of these structures and the comfort of their occupants are also impacted. For these buildings, the vibrations caused by subway operations are a negative factor that cannot be ignored; hence, the structures’ safety and the residents’ comfort are also affected.

Recently, many scholars have been concerned about the influence of tunnel construction on strata and adjacent buildings [2,3,4]. Many studies focus on the specific type of building [5,6,7,8] or surface [9,10,11,12] subjected to the influence of the subway. Using field measurements and numerical simulations, several scholars have investigated the safety of ancient building structures under the impact of traffic-induced vibrations in great detail [13,14,15,16]. Ma et al. [17] established a model of the impact of various types of vibrations on the ancient bell tower and used it to predict the potential impact of the new subway line on the aforementioned structure. Javad et al. [18] classified the subway structural system, the surrounding soil media, and types of cultural and historical structures (CHS). They developed a safe distance prediction model (in the form of practicable graphs) that can be utilized to determine the required minimum metro distances from CHSs. Poovarodom et al. [9] used in situ vibration measurements to determine the intensity of ground vibrations and the attenuation of vibration waves coming from traffic sources and proposed the attenuation curves derived from regression analysis for risk management of the heritage site. Ma et al. [10] proposed a soil–tunnel periodic model with a track slab based on wave transform to simulate the propagation of train-induced vibrations and predict ground vibrations induced by subways, which can be used to predict the areas with the most severe vibration effects and design vibration mitigation measures. Nielsen et al. [11] proposed a hybrid model for predicting ground vibration caused by discrete irregularity of wheels and tracks, showing the effects of wheel plane size and vehicle speed on the maximum wheel–rail vertical contact force and free-field ground vibration. However, there are few studies on the impact of subway-induced vibration on historical and cultural reserves. There are many structures with different shapes in the broad area of the historical and cultural district. In this regard, a comprehensive treatment strategy based on the degree of vibration influence of the building is required; however, there is currently a lack of research in this field.

There are also studies that focus on a specific type of vibration damping measure as the object [19,20,21,22,23,24,25,26,27], some scholars have improved measures such as periodic pile row, metamaterials, and steel spring floating slab track, or adopted new methods to study the vibration reduction effect of these measures, and some scholars have used the life cycle analysis method [28,29] to compare and evaluate several vibration reduction technologies. Kaewunruen et al. [23] were the first to use an effective infinite-boundary train–track–soil coupling model to study the effect of piles on the vibration response of high-speed railways with slab track and concludes that periodic pile rows can significantly reduce the dynamic response of the soil under train vibration, and with the increase in the depth of the pile into the soil, the smaller the dynamic response. Li et al. [25] were the first to investigate the surface vibration mitigation using seismic metamaterial (SMM) barriers in high-speed railways by establishing a 3D coupled train–track–soil model and concluded that the SMM interferes with the propagation path of dynamic waves and attenuates the vibration acceleration in the SMM region. Zhao et al. [27] proposed to use a nonlinear quasizero-stiffness (OZS) vibration isolator to improve the low-frequency vibration damping effect of steel spring floating slab track (FST). The nonlinear characteristics of the vibration isolator and its influence on the dynamic response of FST were analyzed by establishing a dynamic coupling model of QZS-FST, and the key parameters of the QZS vibration isolator were optimized. Kaewunruen et al. [28] conducted a 50-year life cycle analysis of vibration damping methods such as geosynthetics, metamaterials, and foundation improvement, and concluded that geogrids in combination with foundation improvement techniques are economical and effective vibration damping methods. In summary, these studies often focus on a specific vibration reduction method [30]. In practice, measures can be taken in three stages: vibration generation, vibration propagation, and vibration effects on the structure. For various engineering backgrounds, there are significant differences in the effectiveness of these measures [20]. Hence, for cultural protection areas with buildings of different shapes, it is necessary to evaluate the effectiveness of various vibration reduction techniques by considering the engineering background in the area.

In response to this demand, the whole Fayuan Temple historic and cultural reserve is taken as the research object in this paper, large-scale in situ tests are conducted on the surface and buildings of the historic and cultural reserve. Based on the test results, the building’s impact is determined, the influence area is divided according to the impact degree, and the block is designated appropriately. The three vibration stages, namely, active vibration isolation of the vibration source, cutting the vibration propagation channel, and passive vibration isolation of the vibration-affected object, are chosen and used to study the impacts of vibration reduction. Finally, a comprehensive vibration reduction scheme based on regional division is proposed. The vibration zoning method proposed in this paper and the comprehensive vibration reduction scheme based on the vibration zoning can be applied to the vibration reduction and isolation of other similar historical and cultural reserves.

## 2. Problem Background

The Fayuan Temple historic and cultural reserve is located in the city center of Beijing, and its ground is divided into artificial accumulation layer and Quaternary sedimentary layer in the depth range of 21 m disclosed by ground investigation, which mainly consists of gravelly soil, miscellaneous fill, pulverized soil, and sandy soil.

**Figure 1:**
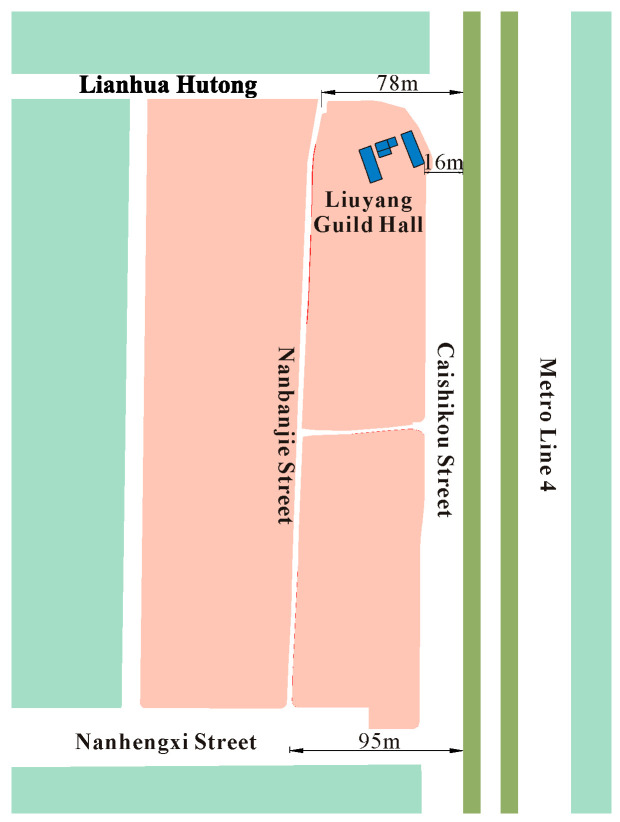
Testing zone.

The Fayuan Temple historic and cultural reserve is affected by vibrations from Metro Line 4. The center-to-center distance of the tracks is 15 m. The horseshoe tunnel, with maximum excavation dimensions of 5.9 m in width and 6.33 m in height, was constructed using the mining method. Metro Line 4‘s north–south route along Caishikou Street is depicted in Figure 1. The distance from the downline’s outer edge to the ancient buildings in the reserve ranges from 16 to 25 m, and the distance from the tunnel to Nanbanjie Hutong varies between 78 and 95 m.

Since Metro Line 4’s inauguration in 2009, the reserve, spanning 13,526 m^2^ and encompassing 435 households, has experienced significant vibration impacts from the subway. The most severe damage is located east of Nanbanjie Hutong. Despite measures such as track polishing and lubrication, vibration reduction is limited and typically lasts only 1 to 2 months. The vibration’s effect on residents has not been fully mitigated.

In order to address this issue, a detailed vibration test was conducted within the reserve to identify vibration-sensitive areas, categorize the vibration-affected zones, and analyze the causes of excessive vibration. Based on this regional division, a comprehensive vibration reduction scheme was proposed, potentially applicable to similar blocks in the future, offering substantial social and economic benefits.

## 3. In Situ Measurement

**Figure 2:**
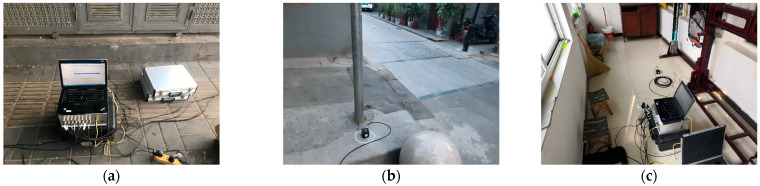
Test equipment and measuring points. (**a**) Test equipment; (**b**) surface vibration measuring point; (**c**) indoor measuring point.

In situ measurements included surface vibration tests and building vibration tests, as illustrated in Figure 2. The test equipment employed a V001 magnetoelectric acceleration sensor, depicted in Figure 2a. Its sensitivity is 0.298 V/(m/s^2^), the measuring range is up to 20 m/s^2^, the frequency range spans from 0.5 to 100 Hz, the resolution varies by gear, ranging from 1 × 10^−8^ to 3 × 10^−6^ m/s^2^, and the operational temperature ranges from −10 to +50 °C. The equipment is equipped with the TDD-16D dynamic signal test and analysis system, which is suitable for high requirements such as modal testing and other testing occasions. The equipment is perfectly suited for low amplitude detection. The sensor was affixed to the measurement point surfaces using glue.

**Figure 3:**
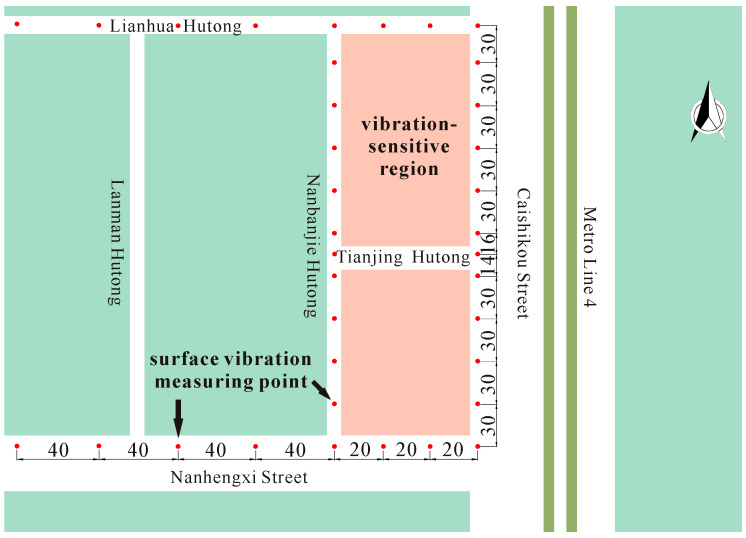
Layout of surface measuring points.

As per the “GB10071-88” Code for “Measurement Methods of Environmental Vibration in Urban Areas” [31] and the “JGJT 170-2009” Code for “Urban Railway Traffic Building Vibration and Secondary Radiated Noise Limits and Measurement Methods Standard” [32], the arrangement of surface measurement points is shown in Figure 3. East–west surface vibration measuring lines were established along Nanheng West Street and Lianhua Hutong, with eight points on each line situated at 20, 40, 60, 80, 120, 160, 200, and 240 m from the subway. Conversely, north–south surface vibration measuring lines along Caishikou Street and Nanbanjie Hutong had points at 0, 30, 60, 90, 120, 150, 180, 210, 240, 270, and 300 m from Nanheng West Street. Additionally, two extra points were positioned at each end of Tianjing Hutong, approximately 134 m from Nanheng West Street.

**Figure 4:**
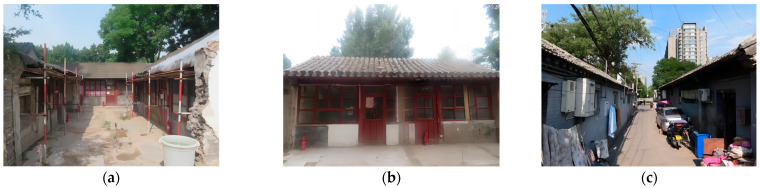
Test equipment and measuring points. (**a**) Liuyang Guild Hall; (**b**) Shaoxing Guild Hall; (**c**) No. 9 Yard, Tianjing Hutong.

The main types of houses in the zone are single-story masonry structures. Six single-story masonry structures with high protection value were selected for vibration testing as follows: Liuyang Guild Hall, No. 2 Yard, Nanbanjie Hutong, No. 108 Yard, Lanman Hutong, No. 7 Yard, No. 9 Yard, Tianjing Hutong, and Shaoxing Guild Hall. The single-layer masonry structure is shown in Figure 4.

**Figure 5:**
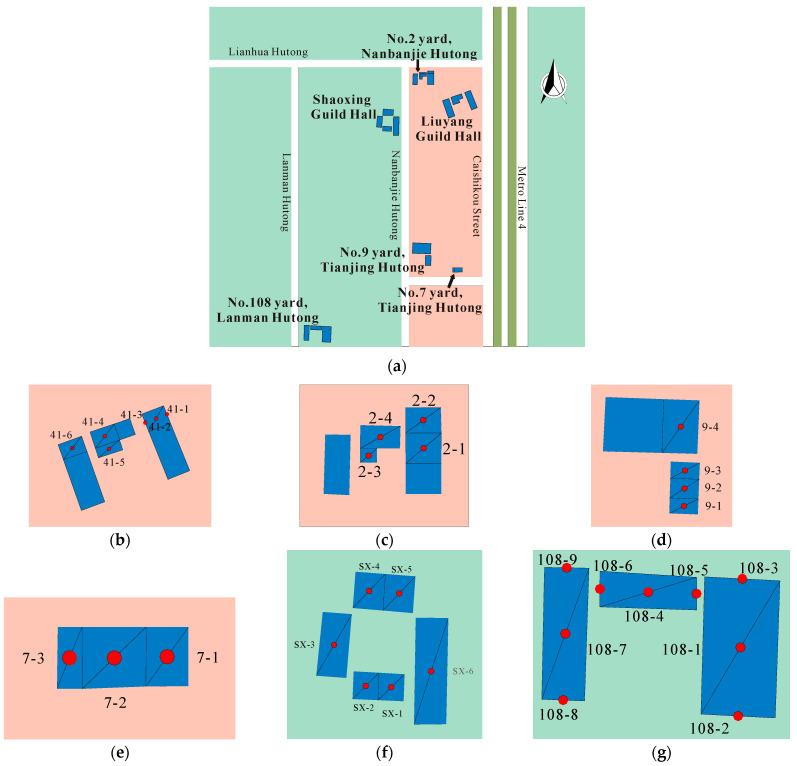
Layout of the indoor measuring points. (**a**) Building location; (**b**) Liuyang Guild Hall; (**c**) No. 2 Yard, Nanbanjie Hutong; (**d**) No. 9 Yard, Tianjing Hutong; (**e**) No. 7 Yard, Tianjing Hutong; (**f**) Shaoxing Huiguan; (**g**) No. 108 Yard, Lanman Hutong.

As per the “GB50355-2018” Code for “Vibration Limits and Measurement Methods of Residential Buildings”, rooms under 20 m^2^ require one measurement point, while rooms over 20 m^2^ need three points [33]. The points’ layout is presented in Figure 5. The distance between the measuring point on the far right of the building and the center line of the train is shown in Table 1.

## 4. Analysis of Vibration Response

### 4.1. Surface Vibration Analysis

**Figure 6:**
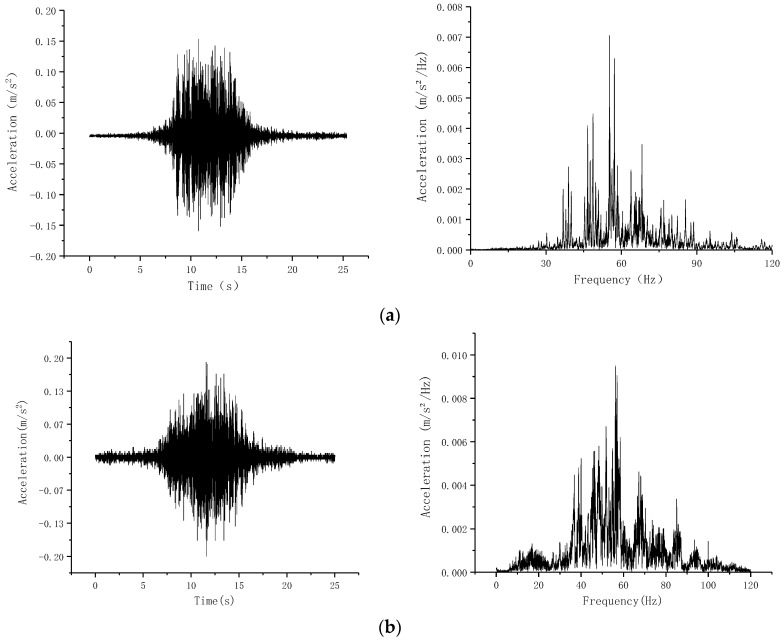
Typical time-history spectrum curve of surface vibration. (**a**) Dynamic response of the subway alone; (**b**) dynamic response of both the subway and the ground traffic.

In order to capture the typical time history of ground vibration as the subway passed, testing occurred from 4 a.m. to 9 a.m. Since most of the measurement point data in the Nanbanjie Hutong are difficult to identify typical spectrum features of subway vibration, only the date to Caishikou Street are analyzed in this section. The spectral analysis results are displayed in Figure 6. These can be categorized into two time frames:

(1) Between 5 a.m. and 6 a.m., the vibrations reflect only the subway’s dynamic response.
(2) Between 7 a.m. and 8 a.m., the vibrations represent the dynamic response of both subway and ground traffic.

Figure 6 illustrates that the time-history vibration curve under the combined influence of subway and ground traffic resembles that under the subway’s sole influence. The spectrum curve reveals that the subway-generated vibrations mainly fall within the 30–90 Hz range, concentrating around 60 Hz. The frequency distribution under subway-only conditions is more focused, with multiple distinct wave peaks, compared to the broader, higher amplitude distribution seen under the combined influence. This analysis indicates that the subway is the primary source of ground vibrations.

Moreover, the time-history vibration curve in Figure 6 shows that the peak vibration acceleration caused by the combined effect is slightly higher than that by the subway alone. The spectrum curve comparison suggests a more uniform frequency distribution under combined action, with larger amplitudes than those caused by the subway alone. Thus, the combined impact of subway and ground traffic is considered more significant in influencing ground vibrations.

Additionally, the vibration propagation law and the vibration acceleration levels at various horizontal distances from the subway were analyzed. The test data from 16 measuring points along the transverse surface vibration measuring line of Lianhua Hutong and Nanheng West Street were collected during periods of dense and non-dense traffic. The effective value of vibration acceleration acquired from the test was converted into a vibration acceleration level according to GB10070-1988, “Urban Area Environmental Vibration Standard” [34]. The vibration acceleration level, abbreviated as VAL, is expressed as follows:

(1) $$\mathrm{VAL}=20\mathrm{lg}\frac{a}{a_{0}},$$

where *a* is the effective value of vibration acceleration in m/s^2^ and *a*_0_ is the effective value of vibration reference acceleration given as *a*_0_ = 10^−6^ m/s^2^.

**Figure 7:**
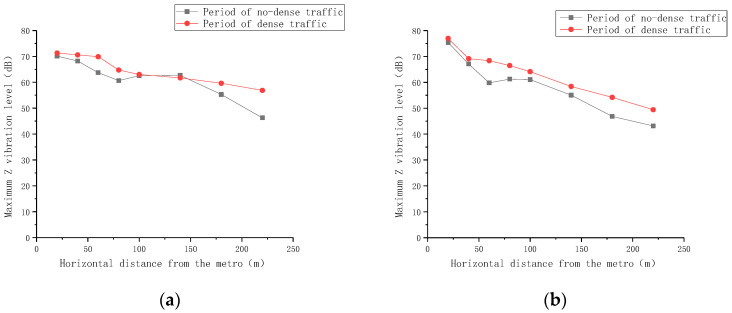
Maximum vertical vibration level curve of subway vibration transmission horizontally. (**a**) Nanheng West Street; (**b**) Lianhua Hutong.

The test results are shown in Figure 7.

From Figure 7, it is evident that the vibration caused by the subway shows a downward trend in the horizontal direction. Furthermore, the vibration produced during the period of dense ground traffic is slightly greater than that during the period of non-dense ground traffic. This observation indicates that the subway is the primary cause of the excessive vibration. Within a 20 m horizontal distance from the line, the maximum vertical vibrations at the measurement point exceed the 70 dB limit. Conversely, the maximum vertical vibration level at the measurement point 100 m from the subway tends to rise, which contradicts the general vibration reduction law. This anomaly can be attributed to the measurement point’s location at the intersection of the measuring line and Nanbanjie Hutong, where there is heavier traffic flow.

### 4.2. Analysis of Building Vibration

**Figure 8:**
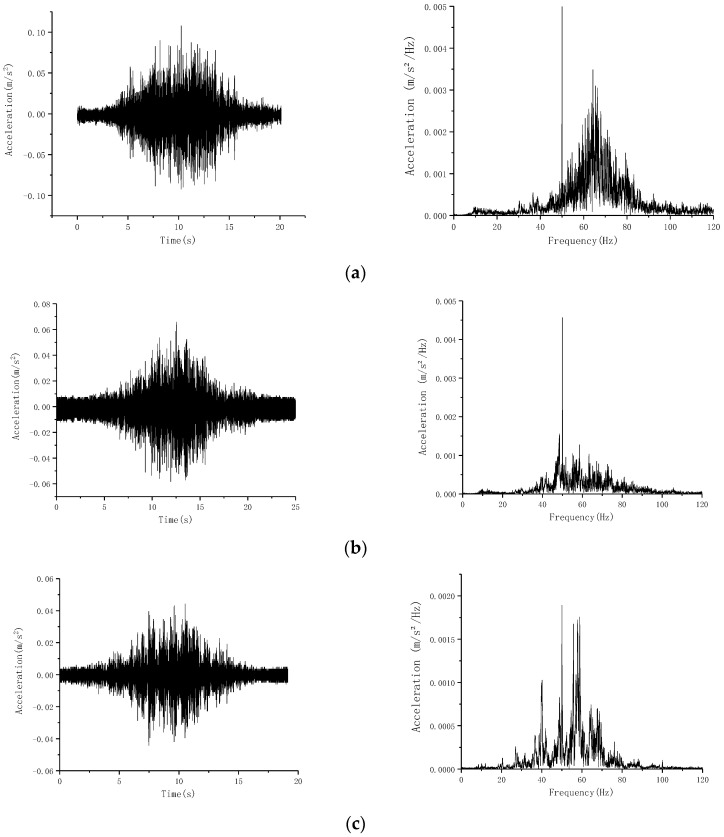
Typical time-history spectrum curve of surface vibration. (**a**) Liuyang Guild Hall; (**b**) No. 7 Yard at Tianjing Hutong; (**c**) No. 9 Yard at Tianjing Hutong.

In the vibration test of the selected six masonry structures, the Liuyang Guild Hall, Tianjing Hutong No. 7 Courtyard, and No. 9 Courtyard were more sensitive to vibration. Therefore, these three buildings were the focus of the analysis. The typical vibration time history and spectrum curve that were obtained are shown in Figure 8.

Figure 8 depicts the vibration responses of the aforementioned buildings. The recorded signals are similar to those at ground measurement points, with the dominant frequency component being 40–80 Hz, where vibrations are mainly caused by the contact between the train and the track [35]. Therefore, the selection of vibration reduction and isolation measures should focus on the weakening or isolation of the high frequency band (40–80 Hz). Meanwhile, the peak vibration acceleration inside the building is in the range of 0.002–0.005 m/s^2^, and the peak vibration acceleration of the surface vibration signal in Caishikou Street recorded in Figure 6b is 0.0097 m/s^2^, indicating that the vibration inside the building is weakened compared with the outdoor surface vibration signal.

And the amplitude of vibration in each building does not vary significantly. Therefore, the maximum vertical vibration of the three buildings with obvious peaks was analyzed, and the results are presented in Table 2, Table 3 and Table 4.

Based on GB10070-1988 “Urban Area Environmental Vibration Standard”, residential structures near the subway have an indoor vibration limit of 67 dB at night and 70 dB during the day [34]. Table 2, Table 3 and Table 4 indicate that among the three buildings, most of the values at the Liuyang Guild Hall and No. 7 Yard in Tianjing Hutong exceed the standards. In contrast, No. 9 Yard in Tianjing Hutong is slightly affected by subway vibration, with few measuring points exceeding the standard. The correlation between the horizontal distance from the subway and the impact of subway vibration on the buildings is inversely proportional. The statistical table shows that the Z vibration levels of the three buildings exhibit little difference between the combined action of the subway and ground traffic and the subway alone. Therefore, the subway is a significant factor causing the buildings’ vibrations to exceed the standard.

### 4.3. Area Division Based on Vertical Vibration Level Distribution

In order to reasonably develop vibration control measures, it is necessary to evaluate the degree of vibration in the vibration affected area and divide the corresponding vibration affected level of the area, so that the active control measures can be selected with a specific target and appropriate control schemes can be adopted.

**Figure 9:**
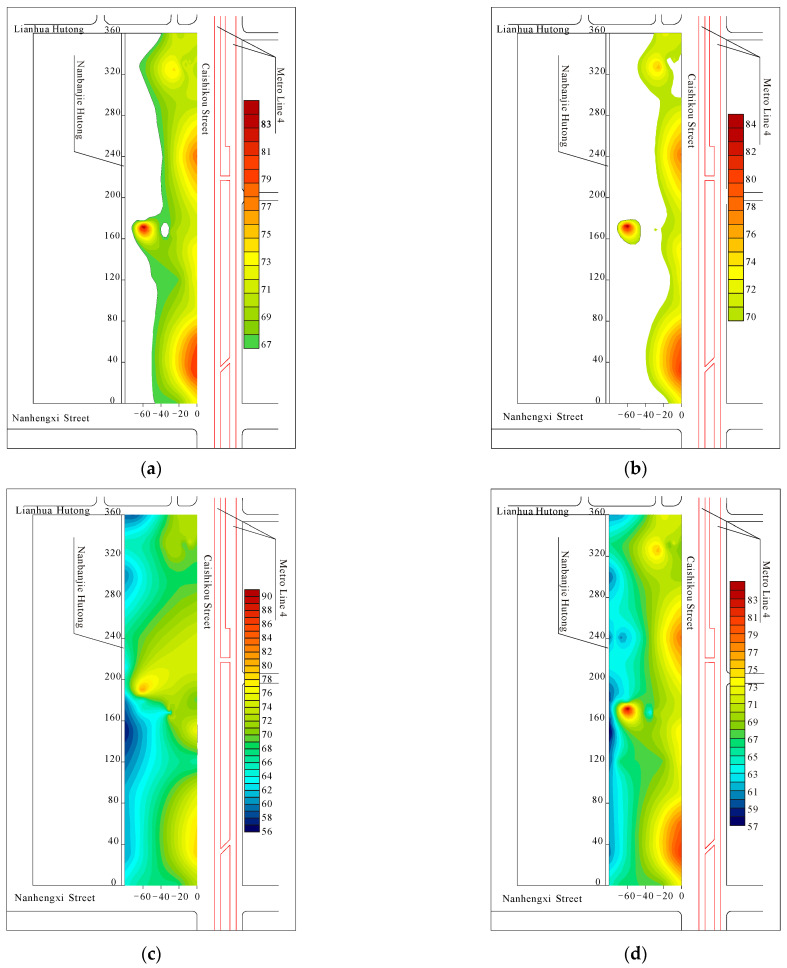
Contour map of vertical vibration level distribution. (**a**) Influence of subway alone; (**b**) influence of subway and ground; (**c**) district with vertical vibration level exceeding 70 dB; (**d**) district with vertical vibration level exceeding 67 dB.

Based on the vibration test results, the horizontal contour map of vertical vibration in the Fayuan Temple historic and cultural reserve was created. The areas where the vertical vibration levels exceed 67 and 70 dB under the combined action of subway and ground traffic were extracted for analysis, as shown in Figure 9.

Figure 9c,d show that the area of excessive vibration is distributed along the west side of Caishikou Street and decreases with increasing distance from Caishikou Street. According to the vertical vibration level distribution in the Fayuan Temple historic and cultural reserve, the reserve is divided into areas of severely excessive vibration, generally excessive vibration, and no excessive vibration. The classification criteria and related areas are presented in Table 5.

**Figure 10:**
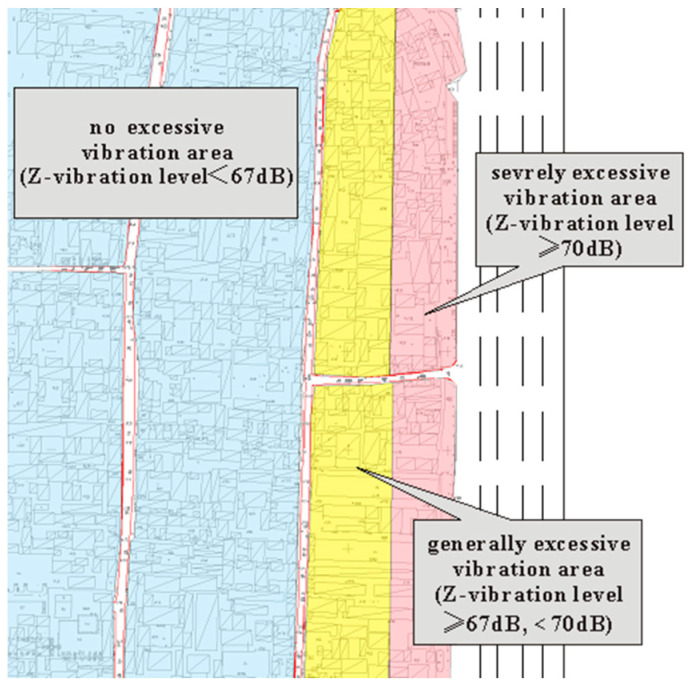
Vibration-affected area.

According to the division in Table 5, the diagram of the vibration influence area was drawn, as shown in Figure 10. It is suggested that measures should be taken to reduce vibration in severely and generally excessive vibration areas.

## 5. Study on Vibration Reduction Measures

Currently, the common vibration isolation measures are divided into three categories as follows: active vibration isolation, cutting vibration propagation paths, and passive vibration isolation. Common active vibration isolation measures include grinding rail, installing damping fastener, laying steel spring floating plate rail and so on. The common measures to cut off the vibration propagation path are air ditch isolation, continuous wall isolation, periodic pile isolation, etc. [36,37,38].

**Figure 11:**
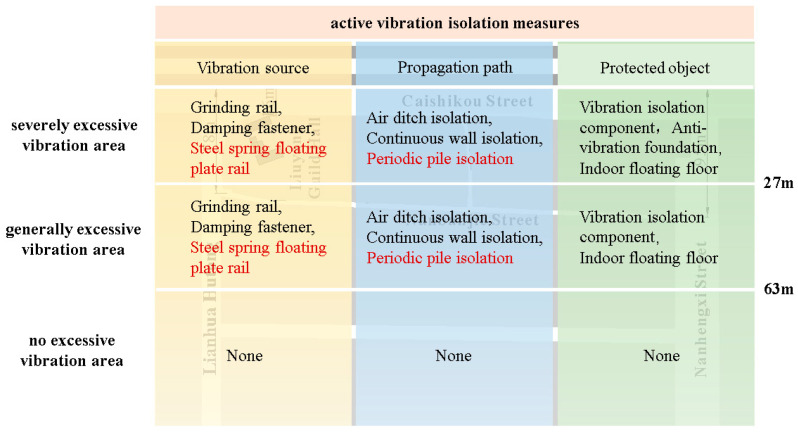
Affected area and its control measures.

In order to reasonably formulate vibration control measures, reasonable control measures are selected in different regions based on the regional division in Section 4, as shown in Figure 11. This chapter mainly introduces three kinds of vibration reduction measures, namely, steel spring floating plate track vibration reduction, periodic pile row vibration reduction, and building anti-vibration reinforcement or renovation, and carries out numerical simulation calculations for the vibration reduction effect of steel spring floating plate track vibration reduction and periodic pile row vibration reduction for the severely exceeding vibration in Fayuan Temple historic and cultural reserve.

### 5.1. The Dynamic Coupling Model of Vehicle–Track–Tunnel–Stratification–Structure

#### 5.1.1. Model Building

**Figure 12:**
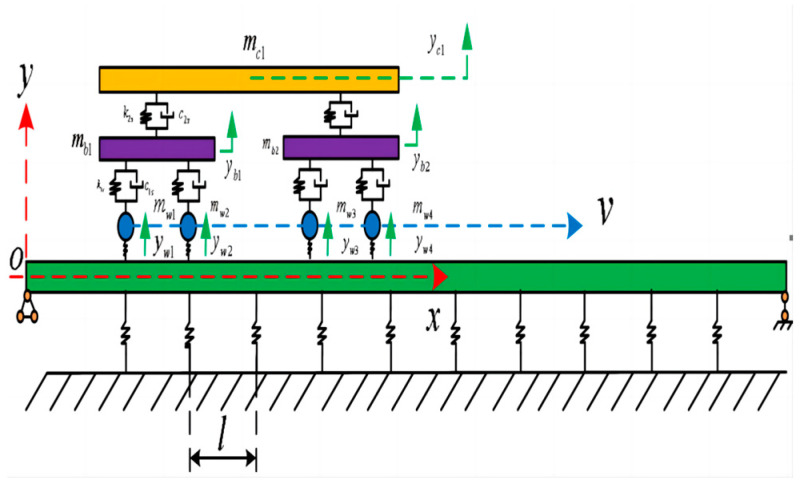
Coupling model of train-track.

According to the relative position between Liuyang Guild Hall and Metro Line 4, a dynamic coupling model of vehicle–track–tunnel–stratification–structure was established. The first part of this model is the vehicle–rail coupling model, as shown in Figure 12. This model comprises a train model, a track model, and a wheel–rail coupling relationship. The train model consists of a body, two bogies, and four wheelsets. The track interacts with the foundation through fasteners, which are considered discrete supported Euler beams [39]. Hertz’s nonlinear contact theory simulated the elastic contact between the wheel and rail.

**Figure 13:**
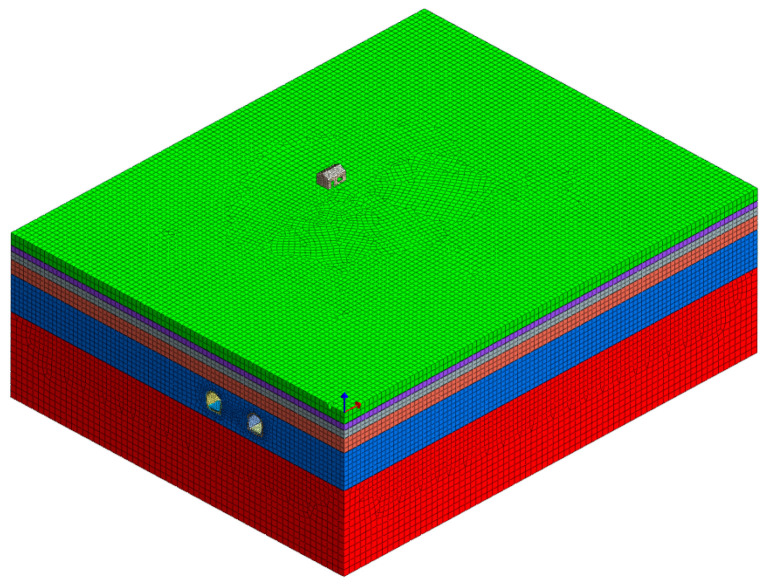
Tunnel–stratum–structure finite element model.

The second part, a tunnel–stratum–structure model, is depicted in Figure 13. This model was created using the three-dimensional finite element software MIDAS-GTS 2019. Its dimensions are 150 × 100 × 50 m (length × width × height), with the minimum mesh size being 0.3 m. Rayleigh’s linear group method calculated the model damping. The viscoelastic boundary conditions were implemented using the ground surface spring element.

#### 5.1.2. Materials Parameters

1. Soil parameters

Based on an actual measured survey report, the modeled stratigraphy was divided into six layers according to soil conditions, as shown in Table 6.

2. Tunnel and ground building structure parameters

The material parameters for the tunnel and roadbed were sourced from the Beijing subway tunnel. The tunnel is made of No. C50 concrete, and the roadbed of No. C30 concrete. The parameters for the tunnel, track, and ground building are presented in Table 7.

3. Rayleigh damping parameters

The damping matrix in the model was calculated using Rayleigh’s linear combination method, assuming that the damping matrix of the system is a linear combination of the mass matrix [M] and stiffness matrix [K].

The expression for the sum of Rayleigh damping constants is as follows:

[C] = α [M] + β [K],(2)

(3) $$\alpha =\frac{2\omega_{i}\omega_{k}}{\omega_{i}+\omega_{k}}\zeta ,$$

(4) $$\beta =\frac{2}{\omega_{i}+\omega_{k}}\zeta ,$$

where [C] is 0.03, $\omega_{i\;}\mathrm{and}\omega_{k}$ are the vibration frequencies of the two modes of the system; ζ is the damping ratio of the system and the angular frequency of the two modes of the system, α is 0.2370 and β is 0.0038.

#### 5.1.3. Materials Parameters

In order to avoid vibration reflection at the truncation boundary, a spring-damping absorption boundary was set. In MIDAS/GTS 2019, the spring stiffness coefficient adopts the foundation reaction coefficient, and the damping coefficient is determined by the following formula:

(5) $$\mathrm{Normal}\;\mathrm{boundary}\text{:}\;C_{\mathrm{fi}}=c_{\mathrm{pi}}A_{i},$$

(6) $$\mathrm{Tan}\mathrm{gential}\;\mathrm{boundary}\text{:}\;C_{\mathrm{qi}}=c_{\mathrm{si}}A_{i},$$

where $c_{\mathrm{pi}}\mathrm{and}c_{\mathrm{si}}$ are unit area damping constants of compression wave and shear wave, respectively; A_i_ is the area represented by the boundary point i.

(7) $$c_{\mathrm{pi}}=\rho_{i}\sqrt{\frac{\lambda +2G}{\rho_{i}}\;},$$

(8) $$c_{\mathrm{si}}=\rho_{i}\sqrt{\frac{G}{\rho_{i}}},$$

where $\lambda =\frac{\mathrm{vE}}{\left(1+v\right)\left(1-2v\right)}$; G is the shear elastic modulus; ρ is the density of the material; E is the elastic modulus.

#### 5.1.4. Validation of Numerical Models

**Figure 14:**
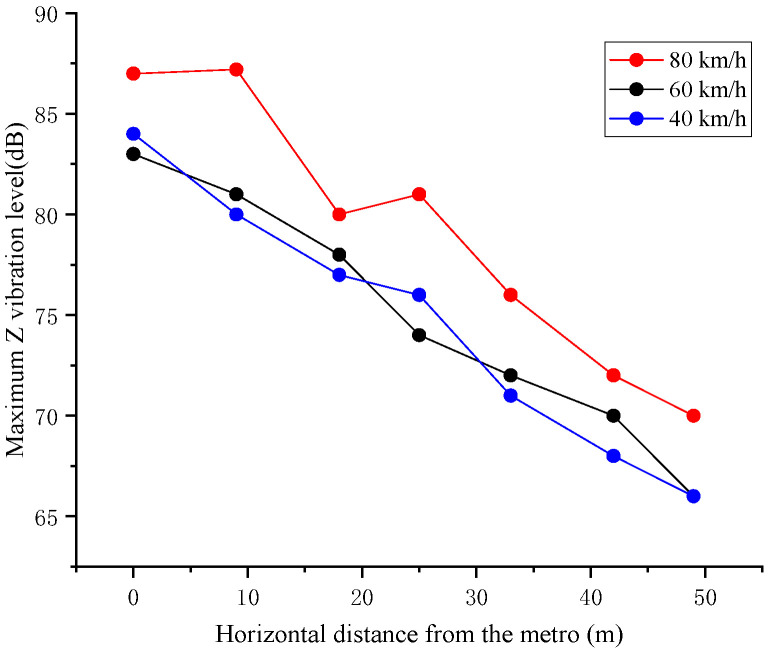
Maximum Z vibration level curve of subway vibration transmission horizontally.

In order to verify the accuracy of the numerical models and analysis method, the structural part of the tunnel–stratum–structure model is deleted to form the tunnel–soil model, and the ordinary track wheel-rail force shown in Figure 14 is applied for calculation. The maximum Z vibration level curve of subway vibration transmission horizontally are extracted at different train operating speeds, as shown in Figure 14, and compared with the measured Z vibration level data on the surface, as shown in Table 8.

The maximum Z-levels of 20 m and 40 m from the metro obtained by numerical calculation are close to the measured results. Due to the uncontrollable speed of the subway in the measurement, there is a difference between the two results, the error range meets the calculation requirements, and the model can be used to carry out subsequent calculations.

### 5.2. Steel Spring Floating Slab Track

This railway structural system was first used in Germany in 1965. Due to its excellent vibration and noise reduction performance, it is frequently utilized in urban rail transit. This track type is currently used by numerous subway lines in Beijing, including the special sections of Lines 4, 5, 9, 10, and 13 of the Beijing Metro.

**Figure 15:**
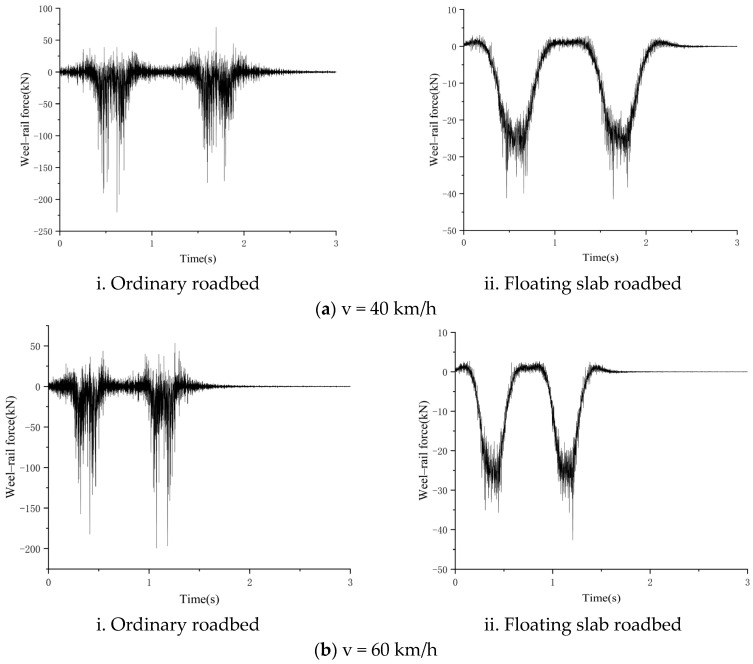

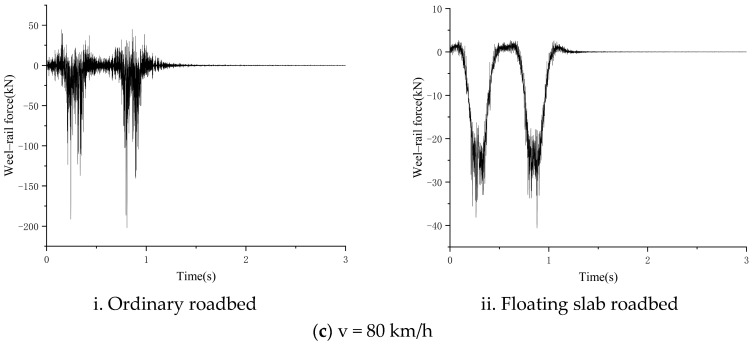
Calculation results of wheel–rail force.

The wheel–rail force (fastener response force) of the steel spring floating slab track and regular ballast bed track at v = 40, 60, and 80 km/h was determined using MATLAB (version 9.10) and the above-mentioned vehicle–track coupling model. The outcomes are displayed in Figure 15.

The vibration responses of the standard section and the extension section of the tunnel in this study were simulated under three different operating speeds. The standard section simulates the train intersection condition, and the train runs in opposite direction at constant speed in the left and right line tunnel. The extended section simulates the extended section condition of the right line, and the train travels at a constant speed in the tunnel of the left line. The calculation conditions are shown in Table 9.

The wheel–rail force in the calculations was input into the tunnel–stratum–structure model, whereas the vibration response of the sensitive building was obtained when the steel spring floating slab track was used or not. The track state of the plate spring floating plate was then compared with the common track state. The maximum vertical vibration level represented the vibration response, and the results are shown in Table 10.

It can be inferred from Table 8 that the steel spring floating slab track has a significant damping effect. The maximum vertical vibration level at measuring points in sensitive buildings was reduced by 6.93 to 10.81 dB (reduction rate is 9.9% to 14.0%).

### 5.3. Periodic Pile Row

**Figure 16:**
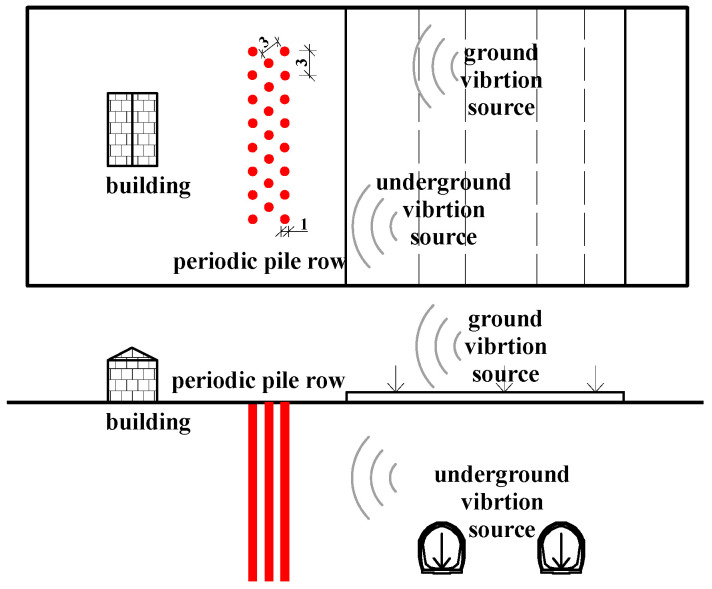
Arrangement plan of periodic pile row.

The common configuration of multi-row pile construction features equal spacing, which prevents the propagation of vibration waves within the band gap frequency range through such periodic structures. Thus, periodic pile rows can control and isolate vibrations of specific frequencies [40,41,42]. The staggered pile row used in this study, with a 3 m core spacing and a 1 m pile diameter, comprises three rows, as shown in Figure 16.

**Figure 17:**
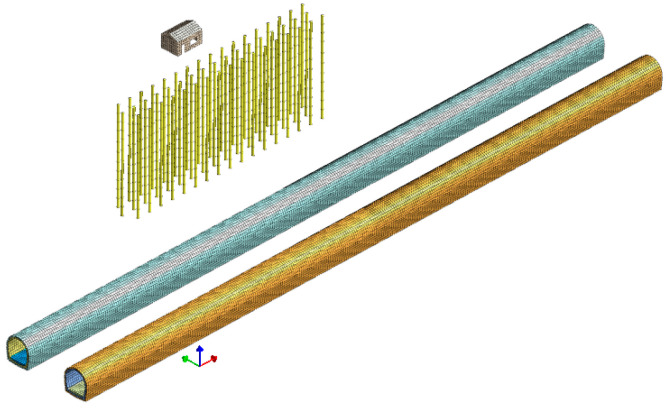
Row piles finite element model and tunnel-stratum-structure model.

The row piles’ finite element model and the tunnel–stratum–structure model are based on the aforementioned parameters. The grid parameters, damping conditions, boundary conditions, and wheel–rail forces are consistent with the above description. The model is depicted in Figure 17.

As Table 11 indicates, 12 conditions were established to simulate the vibration response at various speeds, tunnel sections, and vibration-damping measures. Each condition simulates the encounter of two trains traveling at a constant speed.

The vibration response of a train passing under piling was calculated and compared with that under no measure. The maximum vertical vibration level represents the vibration response, with results shown in Table 12.

Table 12 reveals that the periodic pile row significantly reduces vibrations affecting sensitive buildings in this area. The maximum vertical vibration level at measuring points in sensitive buildings decreased 6.11 to 9.98 dB (a reduction rate from 8.7% to 12.9%).

### 5.4. Anti-Vibration Reinforcement or Reconstruction of the Building

For the excessively vibrating area of Fayuan Temple historic and cultural reserve, targeted reinforcement or reconstruction measures can be formulated for the affected houses. These specific techniques include the following:

- Foundation vibration isolation

The primary steps involve demolishing the original structure, installing vibration isolation devices on the foundation, and reconstructing the superstructure. The vibration isolation component serves as the elastic link between the building and the foundation, reducing vibrations by controlling the transmission rate of the isolation system. Depending on the building’s form, isolation components may include steel spring isolators, rubber isolators, or isolation pads.

- Foundation anti-vibration

After demolishing the original building, a new building with an anti-vibration foundation was designed and constructed. Typical measures include pile foundations, raft foundations, and new basements. The ground floor’s vibration input was reduced due to the altered load transfer path, which lowered the building’s shaking intensities.

- Indoor floating floor

The indoor floating floor reduces vibration by utilizing isolation components and controlling the isolation system’s transmission rate. A concrete floor slab was laid over the structural floor slab, supported by vibration-isolation components. The floating floor’s thickness is generally from 50 to 100 mm, with a 30 to 50 mm gap between it and the structural floor. Steel spring vibration isolators are commonly used as the vibration isolation element, offering effective vibration isolation.

## 6. Vibration Reduction Scheme

Considering the effects, feasibility, and economy of these measures, their advantages and disadvantages are compared, as shown in Table 13.

Taking into account the site conditions, construction difficulty, and project cost, the periodic pile row was preferable to internal subway reconstruction and building reinforcement or reconstruction, making it the priority vibration reduction scheme.

In order to ensure reasonable and economic vibration reduction and isolation and reduce the impact of vibration on the cultural protection area, necessary measures shall be taken to control the impact of vibration within the permissible range. According to in situ measurements and the study of various vibration reduction techniques, comprehensive multi-aspect solutions were proposed for buildings located in different affected areas.

- Buildings in the severely excessive standard area (vertical vibration level ≥ 70 dB):
  ➀ If feasible, a periodic pile row should be the primary measure to reduce vibration in the Fayuan Temple historic and cultural reserve, with anti-vibration reinforcement or reconstruction of the building as supplementary measures. Periodic pile rows significantly reduce vibration transmission when used as the primary strategy, while targeted anti-vibration reinforcement or reconstruction can address specific local areas or cultural buildings with serious over-standard issues.
  ➁ If periodic pile rows cannot be constructed, residents should be relocated, and homes converted from residential to commercial use.
  ➂ If periodic pile driving is not feasible and residents cannot be relocated, the vibration isolation foundation, vibration resistance foundation, or indoor floating floor can be used to transform or reinforce the building.
- Buildings in the generally excessive standard area (vertical vibration level ≥ 67 dB, <70 dB):
  ➀ If feasible, the use of a periodic pile for vibration isolation can meet human comfort requirements.
  ➁ If a periodic pile row cannot be completed, the building’s foundation or main body can be reorganized and reinforced to enhance its anti-vibration performance.

## 7. Conclusions

Most of the previous studies only focus on the surface or specific types of buildings, but lack research on the vibration law and vibration mitigation measures of the whole area of vibration. In order to study the extent of vibration impact in the Fayuan Temple historic and cultural reserve and to determine an appropriate vibration reduction scheme, comprehensive testing of the surface and buildings in this area was conducted. Based on the in situ test results, the reserve was categorized into three zones with varying levels of vibration, determined by the maximum vertical vibration level. For regions with excessive vibration, a dynamic coupling model encompassing vehicle, track, tunnel, stratum, and structure was developed. The effectiveness of the wire spring floating slab track and periodic pile row in mitigating vibration was validated. A comprehensive solution for these regions was formulated based on this research. The main conclusions of this paper are as follows:

(1) The in situ test indicated that the vibration levels of several buildings in the reserve exceeded the standard limits. Analysis of the typical time-history spectrum curve and the maximum vertical vibration level suggested that the subway was the primary source of excessive vibration in this area. The area experiencing excessive vibration was mainly situated between Caishikou Street and Nanbanjie Hutong, with approximately 40% of the areas exceeding the daylight control level of 70 dB and 60% surpassing the nighttime control standard of 67 dB. Based on the vibration test findings, the reserve was segmented into zones of extremely excessive vibration, typically excessive vibration, and non-excessive vibration.
(2) The control ideas of vibration impact from active vibration isolation at the source, interruption of the vibration propagation path, and passive vibration isolation of the affected object, and the numerical simulation is adopted to study the steel spring floating slab track and periodic pile, and the results show that the steel spring floating plate rail and the periodic pile row can significantly reduce the maximum Z vibration level of the sensitive building, and the reduction ranges are 6.93–10.81 dB and 6.11–9.98 dB, and the reduction rates are 9.9–14.0% and 8.7–12.9%, respectively.
(3) A comprehensive scheme for the Fayuan Temple historic and cultural reserve Temple is proposed by considering the vibration regional division, numerical simulation results, the importance of the buildings in the historic and cultural reserve, and the operability of the construction. Periodic pile row should be considered the primary control measure, and anti-vibration reinforcement or reconstruction of buildings should serve as a supplementary measure in the areas with severely excessive standards. For buildings in generally excessive standard areas, employing periodic pile vibration isolation can satisfy the needs of building protection and human comfort requirements.
(4) In the process of measurement in this study, the comprehensive schemes mentioned in this paper have not been taken in the protected area, so we cannot test the vibration reduction effect of the comprehensive vibration reduction scheme in this area. After the comprehensive vibration reduction scheme is adopted in this area, we will carry out in situ testing and compare it with this study.

It is also noted that this study proposes a comprehensive vibration reduction scheme based on the vibration area division in the context of the Fayuan Temple historic and cultural reserve. This idea can be used to formulate comprehensive vibration reduction schemes for other similar cultural reserves, and in the future, the method can be studied and improved in depth by taking into account the effect of the implementation of this comprehensive vibration reduction scheme in the Fayuan Temple historic and cultural reserve.

## Figures and Tables

**Table 1:** The distance between the measuring point on the far right of the building and the center line of the train (m).

Name of Building	Distance (m)
Liuyang Guild Hall	20 m
No. 2 Yard, Nanbanjie Hutong	52 m
No. 9 Yard, Tianjing Hutong	55 m
No. 7 Yard, Tianjing Hutong	28 m
Shaoxing Huiguan	105 m
No. 108 Yard, Lanman Hutong	160 m

**Table 2:** Vertical vibration of Liuyang Guild Hall.

Number of Measuring Points	Liuyang Guild Hall			
	Subway Alone		Both the Subway and the Ground Traffic	
	Vibration (dB)	Whether Overrun	Vibration (dB)	Whether Overrun
41-1	74.74	Yes	72.29	Yes
41-2	69.84 (≈70)	Yes	68.14	NO
41-3	67.77	NO	68.05	NO
41-4	76.24	Yes	75.82	Yes
41-5	73.60	Yes	75.19	Yes
41-6	69.73 (≈70)	Yes	73.41	Yes

**Table 3:** Vertical vibration level of No. 7 Yard, Tianjing Hutong.

Number of Measuring Points	No. 9 Courtyard of Tianjing Hutong			
	Subway Alone		Both the Subway and the Ground Traffic	
	Vibration (dB)	Whether Overrun	Vibration (dB)	Whether Overrun
9-1	66.26	No	87.39	Yes
9-2	64.79	No	65.07	No
9-3	62.31	No	63.19	No
9-4	82.40	Yes	63.57	No

**Table 4:** Vertical vibration level of No.9 Yard, Tianjing Hutong.

Number of Measuring Points	No. 9 Courtyard of Tianjing Hutong			
	Subway Alone		Both the Subway and the Ground Traffic	
	Vibration (dB)	Whether Overrun	Vibration (dB)	Whether Overrun
9-1	66.26	No	87.39	Yes
9-2	64.79	No	65.07	No
9-3	62.31	No	63.19	No
9-4	82.40	Yes	63.57	No

**Table 5:** Division of area with excessive vibration.

Name	Classification Standard	Area (The West Side of Caishikou Street)
Severely excessive vibration area	Vertical vibration level ≥ 70 dB	Distance < 27 m
Generally excessive vibration area	Vertical vibration level ≥ 67 dB, <70 dB	Distance ≥ 27 m,<63 m
No excessive vibration area	Vertical vibration level < 67 dB	Distance ≥ 63 m

**Table 6:** Soil parameters.

Number	Soil Name	Density (kg/m^3^)	Dynamic Elastic Modulus (MPa)	Moving Poisson’s Ratio	Thickness (m)
1	Crushed stone fill	1.7	164	0.17	3.7
2	Plain fill	1.89	235	0.17	1.9
3	Sandy silt	1.92	262	0.16	2.5
4	Silt	2.1	404	0.16	3.5
5	Medium sand	2.15	498	0.16	9.5
6	Silty clay	1.98	374	0.16	28.9

**Table 7:** Tunnel and ground building structure parameters.

Number	Name	Density (kg/m^3^)	Elastic Modulus (MPa)	Poisson’s Ratio
1	Tunnel	2500	3.5 × 10^4^	0.2
2	Brick foundation	1850	3040	0.15
3	Underlay of brick foundation	2360	1.85 × 10^4^	0.2
4	Brick masonry (standard brick)	1850	3040	0.15

**Table 8:** Comparison of maximum Z-vibration level between in situ test and numerical models (dB).

Distance (m)	Z—Level of in Situ Test (dB)		Z—Level of Numerical Models (dB)		
	Nanheng West Street	Lianhua Hutong	40 km/h	60 km/h	80 km/h
20	70	75	76	77	80
40	68	67	70	71	73

**Table 9:** Calculation condition of steel spring floating slab track.

Calculation Conditions	Train Speed (m/s)	Section	Shock-Absorbing Measure
1	40	Standard section	Steel Spring Floating Slab Track
2	40	Standard section	No
3	40	Large section	Steel Spring Floating Slab Track
4	40	Large section	No
5	60	Standard section	Steel Spring Floating Slab Track
6	60	Standard section	No
7	60	Large section	Steel Spring Floating Slab Track
8	60	Large section	No
9	80	Standard section	Steel Spring Floating Slab Track
10	80	Standard section	No
11	80	Large section	Steel Spring Floating Slab Track
12	80	Large section	No

**Table 10:** Maximum Z vibration level of buildings (unit: dB).

	Speed (km/h)	40	60	80
Vibration Reduction Scheme				
Standard section without shock-absorbing measure		70.70	76.65	79.64
Standard section with steel spring floating slab track		62.51	65.95	71.64
Large section without shock-absorbing measure		66.59	70.03	79.59
Large section with steel spring floating slab track		59.66	63.09	68.78

**Table 11:** Calculation condition of periodic pile row.

Calculation Conditions	Train Speed (m/s)	Section	Shock-Absorbing Measure
1	40	Standard section	Periodic pile row
2	40	Standard section	No
3	40	Large section	Periodic pile row
4	40	Large section	No
5	60	Standard section	Periodic pile row
6	60	Standard section	No
7	60	Large section	Periodic pile row
8	60	Large section	No
9	80	Standard section	Periodic pile row
10	80	Standard section	No
11	80	Large section	Periodic pile row
12	80	Large section	No

**Table 12:** Maximum vertical vibration level of buildings (unit: dB).

	Speed (km/h)	40	60	80
Vibration Reduction Scheme				
Standard section without shock-absorbing measure		70.70	76.65	79.64
Standard section with periodic pile row		63.33	66.77	72.46
Large section without shock-absorbing measure		66.59	70.03	79.59
Large section with periodic pile row		60.48	63.92	69.61

**Table 13:** Comparison of vibration reduction schemes.

Serial Number	Control Scheme	Advantages	Disadvantages
1	Internal vibration reduction reconstruction of subway	The issue of excessive vibration in cultural reserves can be fully resolved at the source, and this has a significant impact on governance.	1. Metro Line 4 carries a sizable volume of passengers. To close the subway and renovate it is unrealistic. 2. The small operating window and lengthy building cycle make it challenging to guarantee construction quality, which can impact train operation.
2	Periodic pile row	1. The periodic pile row is constructed on the ground, which is not affected by the subway operation. The process of construction is quick and simple. 2. The issue of excessive vibration in cultural reserves can be a fully resolved vibration propagation path.	The ground traffic around the cultural protection area will be affected by the construction, resulting in traffic congestion.
3	Anti-vibration reinforcement or reconstruction of the building	The anti-vibration reinforcement or reconstruction of the building can be completed simultaneously.	The majority of the structures in the cultural reserve have shaky foundations and a lengthy history; hence, certain rebuilding efforts may have an adverse effect on structural performance.

## Data Availability

Data are contained within the article.
